# Aqueous Two-Phase Interfacial Assembly of COF Membranes for Water Desalination

**DOI:** 10.1007/s40820-022-00968-5

**Published:** 2022-11-09

**Authors:** Hongjian Wang, Jiashuai Zhao, Yang Li, Yu Cao, Ziting Zhu, Meidi Wang, Runnan Zhang, Fusheng Pan, Zhongyi Jiang

**Affiliations:** 1 Key Laboratory for Green Chemical Technology of Ministry of Education, School of Chemical Engineering and Technology, Tianjin University, Tianjin, 300072 People’s Republic of China; 2 Haihe Laboratory of Sustainable Chemical Transformations, Tianjin, 300192 People’s Republic of China; 3 Department of Chemistry, Tianjin Key Laboratory of Molecular Optoelectronic Science, Tianjin University, Tianjin, 300072 People’s Republic of China; 4 Joint School of National University of Singapore and Tianjin University, International Campus of Tianjin University Binhai New City, Fuzhou, 350207 People’s Republic of China

**Keywords:** Covalent organic framework membranes, Aqueous two-phase, Interfacial polymerization, Molecular separation, Water desalination

## Abstract

Aqueous two-phase system features with ultralow interfacial tension and thick interfacial region, affording unique confined space for membrane assembly. Here, for the first time, an aqueous two-phase interfacial assembly method is proposed to fabricate covalent organic framework (COF) membranes. The aqueous solution containing polyethylene glycol and dextran undergoes segregated phase separation into two water-rich phases. By respectively distributing aldehyde and amine monomers into two aqueous phases, a series of COF membranes are fabricated at water–water interface. The resultant membranes exhibit high NaCl rejection of 93.0–93.6% and water permeance reaching 1.7–3.7 L m^−2^ h^−1^ bar^−1^, superior to most water desalination membranes. Interestingly, the interfacial tension is found to have pronounced effect on membrane structures. The appropriate interfacial tension range (0.1–1.0 mN m^−1^) leads to the tight and intact COF membranes. Furthermore, the method is extended to the fabrication of other COF and metal–organic polymer membranes. This work is the first exploitation of fabricating membranes in all-aqueous system, confering a green and generic method for advanced membrane manufacturing.

**Supplementary Information:**

The online version contains supplementary material available at 10.1007/s40820-022-00968-5.

## Highlights

- All-aqueous phase approach to membrane fabrication is invented for the first time.
- Interfacial tension of water-water interface has pronounced effect on the evolution of membrane structures.
- Covalent organic framework membranes through aqueous two-phase interfacial assembly exhibit high water desalination performances.

**Supplementary Information:**

The online version contains supplementary material available at 10.1007/s40820-022-00968-5.

## Graphical abstract

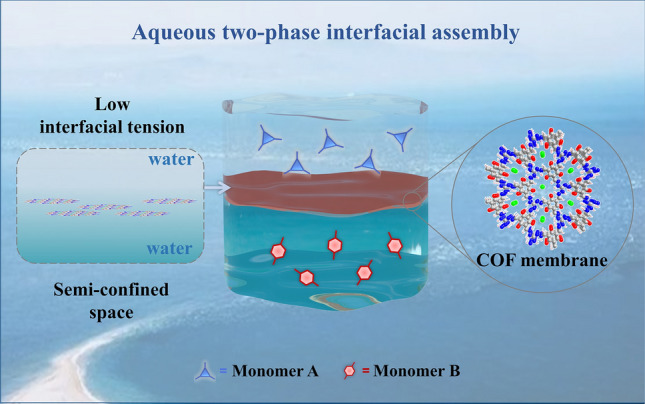

## Introduction

Membrane materials are the chips of membrane technology, which determine the selectivity, permeation flux and operation stability of membranes in diverse chemical separations [1–6]. Currently, most membrane fabrication methods are based on organic solvents and thus employ large amounts of organic solvents in membrane manufacturing [7–10]. It is therefore imperative to exploit organic solvent-free or all-aqueous-phase methods to fabricate high-performance membranes for diverse chemical separations such as water desalination [11–14].

To date, many kinds of membrane fabrication methods have been explored to fabricate membranes with different structures. Among them, interfacial polymerization (IP), solvothermal synthesis, thermally induced phase separation (TIPS) and nonsolvent-induced phase separation (NIPS) have established powerful and most popular platforms for creating membrane structures for different chemical separations [15–18]. IP method and solvothermal synthesis utilize organic solvents to create phase (oil–water or oil–solid) interface, and control the partition-diffusion–reaction and film formation processes of monomers at the interface. The flexibility of monomer selection, reaction design and structural evolution control renders IP method and solvothermal synthesis broad applicability for the fabrication of amorphous polymer (e.g., polyamide (PA)) membranes [19, 20] and crystalline polymer (e.g., covalent organic framework (COF)) membranes [21, 22]. TIPS and NIPS are also well-explored methods for the fabrication of membranes. They use organic solvent to make polymers into homogeneous casting solution and induce the phase (liquid–liquid or solid–liquid) separation to fabricate most polymer-based membranes [23, 24]. However, the methods mentioned above inevitably use organic solvents to create the phase systems of membrane mother solutions, which brings about two important issues to be addressed: (i) rationally developing all-aqueous-phase system to better meet the environmentally benign requirements, and (ii) facilely manipulating interfacial properties to better coordinate the reversible reaction of monomers and the ordered packing of building units.

Aqueous two-phase system (ATPS) is a solvent-free method for hierarchical structures formation and functional material preparation [25, 26]. It stems from an aqueous mixture of two chemically distinct polymers. Under the repulsion interaction of polymers, the phase of mixture spontaneously segregates into two immiscible aqueous phases. Compared with oil–water system, ATPS possesses an all-aqueous composition. The degradable polymer components make ATPS out of the solvent volatilization to environment pollution, which offers a green way for material preparation. Furthermore, ATPS has wide interfacial zone with thickness up to micrometer, and low interfacial tension (0.001–0.1 mN m^−1^) which is two orders of magnitude lower than that of oil–water system [27–30]. These features of ATPS may bring about unique chances for membrane fabrication [31]. Finally, facilely manipulating interfacial properties enables a more favorable synergy between the polymerization reaction of monomers and the controllable packing of building units for the delicate structural evolution of membranes [19, 32].

Here, for the first time, we reported an aqueous two-phase interfacial assembly method to fabricate COF membranes. The ATPS was composed of poly(ethylene glycol) (PEG) and dextran (Dex) that spontaneously demixed into two water-rich phases. With distributing aldehyde and amino monomers into the two aqueous phases, COF-DhTG_Cl_ membranes were fabricated and manipulated by varying the Dex/PEG weight ratio, pH value and reaction time. The resulting membranes exhibited high NaCl rejection of 93.0–93.6% and water permeance reaching 1.7–3.7 L m^−2^ h^−1^ bar^−1^, superior to the state-of-the-art desalination membranes. Interestingly, we found interfacial tension exerted pronounced effect on structural evolution of COF membranes. The ATPS with higher interfacial tension (0.1–1.0 mN m^−1^) led to the tight and intact COF membranes. In sharp contrast, the system with lower interfacial tension (0.001–0.1 mN m^−1^) led to the loose and fragmented COF membranes. Furthermore, our aqueous two-phase interfacial assembly method was extended to the fabrication of other COF membranes and metal organic polymer (metal-organophosphate) membranes.

## Experimental Section

### Reagents and Materials

2,5-dihydroxyterephthalaldehyde (Dh), triaminoguanidinium chloride (TG_Cl_) and 1,3,5-benzenetriamine trihydrochloride (BT_Cl_) were purchased from Jilin Chinese Academy of Sciences—Yanshen Technology Co., Ltd (Jilin, China). PEG (average Mn = 20,000) was purchased from Sanen Chemical Technology (Shanghai) Co., Ltd (Tianjin, China). Dex (Mw = 500,000) was purchased from Nanjing duly Biotechnology Co., Ltd (Nanjing, China). Sodium hydroxide (NaOH), hydrochloric acid (HCl), sodium chloride (NaCl), ferric chloride hexahydrate (FeCl_3_·6H_2_O), 1-octanol, *n*-octane, 1-butanol, dichloromethane, *N,N*-dimethylformamide (DMF), 1,4-dioxane, methanol and ethanol were obtained from Kermel Chemical Reagent Co., Ltd (Tianjin, China). Acetic acid and tetrahydrofuran were obtained from Aladdin Industrial Co., Ltd (Shanghai, China). Phytic acid (PA, 70 wt% in water) was brought from Tianjin Heowns Biochemical Technology Co., Ltd (Tianjin, China). Polyacrylonitrile porous substrates (PAN, MWCO = 100 kDa) were purchased from Lanjing Membrane Technology Co., Ltd (Shanghai, China). Polytetrafluoroethylene porous substrates (PTFE, average pore size = 0.22 μm) were supplied by Haiyan New Oriental Plastic Technology Co., Ltd (Jiaxing, China). Dialysis bag with a MWCO of 50 kDa was supplied by Tianjin Leviathan Technology Co., Ltd (Tianjin, China).

### Preparation of COF Membranes by ATPS

#### Synthesis of ATPS

20 g of PEG (Mw = 20,000) was added into deionized (DI) water (180 mL) to form solution A. 35 g of Dex (Mn = 500,000) was added into DI water (185 mL) to form solution B. When the polymer solutes were completely dissolved, the solution A and B were mixed together and stirred at 25 °C for 12 h. The mixed solution was kept under static condition for 12 h to reach phase equilibrium. It found two obvious phases in the system, where PEG-rich aqueous phase served as the top phase and Dex-rich phase served as the bottom phase. The two phases had a certain thickness (micron level) phase interface area. The ATPS was spontaneously separated to obtain top and bottom aqueous phase. Moreover, other ATPS could be acquired with tuning the relative proportion and total polymer fraction in water, as illustrated in the subsequent supplementary information.

#### Fabrication of COF Membranes

0.004 mmol of Dh and 10 μL NaOH aqueous solution (1 M) were dissolved to 10-mL PEG aqueous phase and sonicated for 30 min to obtain the solution C with pH of about 10. 0.0025 mmol of TG_Cl_ or BT_Cl_ was added to 10-mL Dex aqueous phase and sonicated for 30 min to obtain the solution D. With solution D as the lower phase and solution C as the upper phase, solution C was added to solution D drop by drop. COF-DhTG_Cl_ or COF-DhBT_Cl_ membranes were observed at the water–water interface after the ATPS was standing for 1–7 days. The resulted membranes were transferred with PAN substrates or non-woven fabric substrates and cleaned with DI water and ethanol.

### Characterization

Field emission scanning electron microscopy (SEM, Regulus 8100) was employed to take SEM images of the cross section and surface appearance of membranes. Transmission electron microscopy (TEM, JEOL JEM-F200) was utilized to capture the TEM image, HRTEM image and the selected area electron diffraction patterns of COF membranes. The grazing-incidence wide-angle X-ray scattering (GIWAXS) was applied to obtain the crystalline information of COF powders and COF membranes, using Cu-Kα as the radiation source. Fourier transform infrared spectroscopy (FT-IR, Bruker vertex 70) was used to record the FT-IR spectra of COF powder and reactive monomers, with the condition that wavelength scanning range was 4000–500 cm^−1^ and the step size was 4 cm^−1^. The X-ray photoelectron spectroscopy (XPS) of Thermo Scientific K-Alpha^+^ spectrometer was employed to figure out the element composition of COF powder and COF membranes with the Al-Kα radiation source. The solid-state nuclear magnetic resonance (ssNMR) of 600 MHz JEOL JNM ECZ600R spectrometer was employed to collect the chemical structure information of COF membranes. The Quantachrome Autosorbe-1 analyzer was utilized to carry out the N_2_ adsorption–desorption isotherms at 77 K. The Netzsch TG209 F3 instrument was applied to acquire the thermogravimetric analysis (TGA) curve from 40 to 800 °C with a heating rate of 10 °C min^−1^. The water contact angle (WCA) of DI water on the membrane sample was measured with a JC2000D2M contact angle goniometer (POWEREACH®). Nano-indentation test (Hysitron Ti-Premier, USA) was employed to measure the mechanical properties of membranes. The nano-indentation test was conducted in load control mode. The indentation load was controlled at 1000 μN in all tests, the loading and unloading time was 5 s, and the loading peak remained 2 s.

### Desalination Test

Desalination test was conducted using a home-made nanofiltration apparatus with cross-flow configuration. The feed side was driven by a diaphragm pump and circulated with 1 L of aqueous solution that contained 1,000 ppm NaCl aqueous solution. The operation pressure was tuned from 1.0 to 6.0 bar. Prior to the measurements, the flow of water across membranes was carried under 4.0 bar for 0.5 h to ensure the stabilized water desalination performances of membranes. The conductivity values of permeated water were measured by conductivity meter (DDSJ-308A). The water permeation (*J*_*w*_, L h^−1^ m^−2^ bar^−1^) of permeated water and rejection (*R*, %) were calculated according to Eqs. (1) and (2):

1 $$ J_{w}  = \frac{{\Delta V}}{{\left( {A \times \Delta {\text{t}} \times \Delta {\text{p}}} \right)}} $$

2 $$ R = \frac{{C_{f} - C_{p} }}{{C_{f} }} \times 100\% $$

where Δ*V* (L) was the liquid height in the permeated side, *A* (m^2^) was the effective permeated area, Δ*t* (h) was the permeated time, Δ*p* (bar) was the pressure drop between the feed and permeate sides, *C*_*f*_ (μS cm^−1^) and *C*_*p*_ (μS cm^−1^) were the salinity of the feed and permeate solutions.

## Results and Discussion

### Investigation of Membrane Structures

**Fig. 1:**
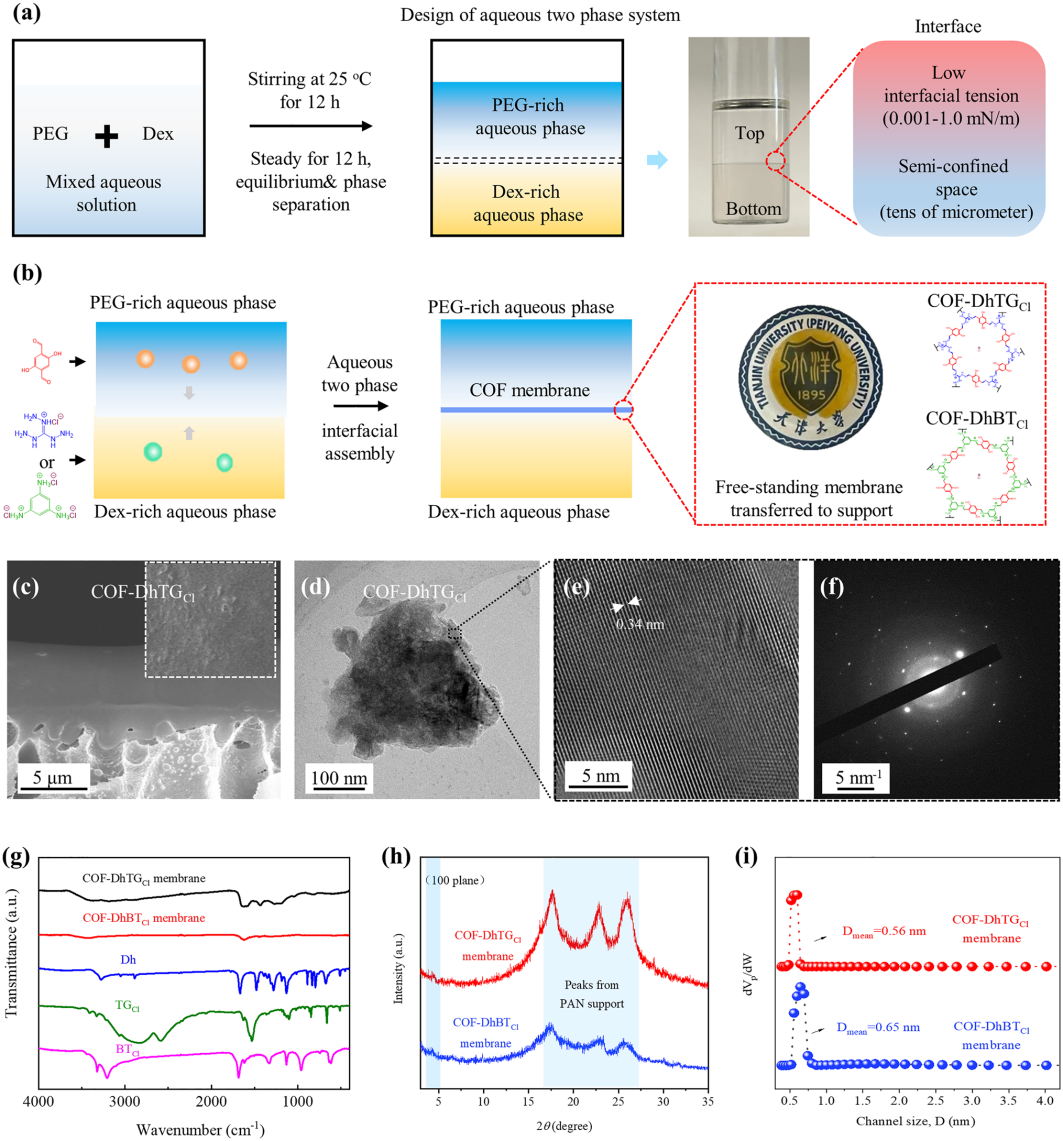
Fabrication and morphology characterization of COF membranes. **a** Designing process for ATPS. **b** Aqueous two-phase interfacial assembly of COF membranes. **c** SEM, **c-d** TEM and **f** SAED measurements on COF-DhTG_Cl_ membrane. **g** FT-IR pattern, **h** GIWAXS pattern and **i** pore size distribution of COF membranes

ATPS was designed with using PEG (Mw = 20,000) and Dex (Mn = 500,000) as aqueous solutes [28, 29]. As shown in Fig. 1a, PEG and Dex were, respectively, added into DI water to form the solution A and B. Subsequently, the solution A and B were mixed together and stirred at 25 °C for 12 h. The mixed solution was kept under static condition for 12 h to reach phase equilibrium. Due to the segregative phase separation, two obvious phases were formed, where PEG-rich aqueous phase served as the top phase and Dex-rich phase served as the bottom phase. Moreover, a sharp interface, featuring with semi-confined space (tens of micrometer) and low interfacial tension (0.001–1.0 mN m^−1^), generated between the top and bottom aqueous phase, demonstrating the as-expected formation of ATPS [26]. COF membranes were fabricated with using aqueous two-phase interfacial assembly method, as shown in Fig. 1b. After isolating the top and bottom phase of ATPS, Dh and NaOH/water were dissolved into PEG-rich solution to obtain top phase, and TG_Cl_ or BT_Cl_ was added into Dex-rich solution to obtain bottom phase. The top phase was slowly dropped onto the bottom phase to proceed interfacial assembly of membrane. Over a period of 3–7 days, the aldehyde and amine monomers diffused oppositely and reaction at aqueous two-phase interface to generate brown COF-DhTG_Cl_ and COF-DhBT_Cl_ membranes (Fig. S1). The free-standing membranes were transferred onto different supports for characterizations and measurements.

SEM and TEM were used to ascertain morphologies of membranes. COF-DhTG_Cl_ membranes exhibited uniform cross-section view structures with thickness around 5.3 μm, and no obvious pinholes or cracks could be found from membrane surface (Fig. 1c). In addition, the surface structures of COF-DhTG_Cl_ membranes were found quite different from those obtained from oil–water interfacial polymerization, which had spherical, crumpled and granular cluster morphologies [21, 33]. This phenomenon was attributed to the unique phase property of ATPS. Owing to similar heat capacity of top and bottom aqueous phases, the heat (from imine formation) generated at interface will be well-dissipated by heating two aqueous phases simultaneously, which rendered flat and tight surface structures [32]. In order to investigate microstructures of membranes, COF-DhTG_Cl_ membranes were sonicated in isopropanol, subsequently transferred onto carbon films. Figure 1d showed lamellar products exfoliated from membranes, illustrating that the units from monomer reaction mostly presented as sheet or plate morphologies for assembling into membranes. Under high resolution, the lamellar products exhibited lattice fringes with large-scale directional alignment (Fig. 1e). The distance between adjacent lattice fringes was measured to be ~ 0.34 nm, matching well with that of π–π stack interlayer. Selected area electron diffraction (SAED) result (Fig. 1f) demonstrated uniform diffraction spots that corresponded to (001) plane of COF-DhTG_Cl_, confirming the crystalline structures of the membranes. Besides, COF-DhBT_Cl_ membrane showed micro-sized thickness and crystalline nature (Fig. S2).

FT-IR spectra clearly demonstrated the formation of the β-ketoenamine framework structures within COF membranes (Fig. 1g). An obvious signal at 1615 cm^−1^, which corresponded to stretch and vibration of C=N, was found in FT-IR spectra, manifesting the successful reaction between Dh and TG_Cl_ (or BT_Cl_) monomers [34–36]. Furthermore, COF membranes did not show specific peaks at 1661 and 3327 cm^−1^ that belonged to the C=O of Dh and N–H of TG_Cl_ and BT_Cl_, respectively. This was due to that the monomers were completely reacted or removed inside COF-DhTG_Cl_ membranes. From XPS result, a peak corresponding to C=N/C–O appeared at 286.8 and 285.5 eV of C 1*s* respectively (Fig. S3), confirming the formation of imine bond within COF membranes. Meanwhile, COF-DhTG_Cl_ membranes showed obvious signal of = ^+^N- at 39.84 eV of N 1*s* which demonstrated the positive charge property of membranes which was in agreement with Zeta potential result (Table S1). Solid-state nuclear magnetic resonance (ssNMR) is shown in Fig. S4. The COF-DhTG_Cl_ and COF-DhBT_Cl_ membranes displayed characteristic peaks of C=N at 150 ppm and carbonyl carbons at 122 ppm and 114 ppm. This result further verified the reaction between Dh and TG_Cl_ (or BT_Cl_) monomers, which formed β-ketoenamine framework structures within COF membranes [37].

GIWAXS illustrated the partial crystalline property of COF membranes. Figure 1h showed intense peak at 2*θ* of 5.2° for COF-DhTG_Cl_, and 4.5° for COF-DhBT_Cl_, which was attributed to the reflection from the (100) plane, respectively, manifesting the (partial) crystalline nature of membranes [38]. The low intensity of (100) plane was similar to the results of powders in the literature. Due to the strong interaction among interlayers, COF-DhTG_Cl_ and COF-DhBT_Cl_ usually exhibited lower crystallinity than non-ionic COFs. In addition, the GIWAXS pattern exhibited three broad signals at 2*θ* of 17°–27° belonging to PAN that supported COF membranes. Due to the high relative intensity of PAN supports, the signal of (001) plane for COF-DhTG_Cl_ was covered up at around 25°–27°. Two-dimensional (2D) synchrotron radiation GIWAXS was used to detect lattice orientation of COF membranes. A broad diffraction ring at around 0.35 and 0.13 Å^−1^ was found in both in-plane and out-plane axis for COF-DhTG_Cl_ and COF-DhBT_Cl_ membrane, respectively (Fig. S5), which indicated that the (100) plane of membrane had no specific orientation [39]. The orange color of (100) plane also confirmed its lower intensity than that of PAN supports with red or pink signal, consistent with results in GIWAXS spectrum. To investigate the channel metric of membranes, N_2_ adsorption analysis was carried out (Figs. 1i and S6). The specific area of membranes based on Brunauer–Emmett–Teller (BET) gave the value of ~ 24 m^2^ g^−1^, which was much lower than that of COF powders. This result was similar to those of other COF membranes [2, 40, 41] and was primarily due to the lamellar structures of COF units within membranes. The high aspect ratio of COF units restricted effective adsorption of N_2_ molecule inside frameworks, leading to the lower surface areas of membranes. Besides, the irregular assembly of COF units that fortified the interactions between adjacent units and resulted in staggered interlamellar pores also contributed to such phenomenon. The inset of Fig. 1i showed a narrow distribution of channel size that concentrated on 0.56 and 0.65 nm for COF-DhTG_Cl_ and COF-DhBT_Cl_ membrane, respectively, less than that of powders (0.7–1.3 nm) [34, 35], further verifying the interlace assembly of COF units could narrow the effective channel size of membranes.

The hydrophilicity of COF-DhTG_Cl_ and COF-DhBT_Cl_ membranes was assessed by water contact angle (WCA) measurement, which were around 62.9° and 69.1°, respectively (Fig. S7). Compared with anionic COF membranes [41, 42] that had a lower WCA of 20°–40°, COF-DhTG_Cl_ membranes possess lager WCA and thus lower hydrophilicity, which may lead to larger mass transfer resistance for water treatment applications. Thermal gravimetric analysis (TGA) indicated the excellent thermal stability of COF membranes (Fig. S8). With the temperature increasing from 40 to 220 °C, the weight loss of membranes was less than 5%, ascertaining the considerable stability of membranes under 220 °C. However, with the temperature exceeding 220 °C, it was found the membranes had a sharp weight loss trend, which demonstrated the frameworks inside membranes had undergone thermodynamic decomposition. Furthermore, to expand the applications, aqueous two-phase interfacial assembly was also used to fabricate metal-organophosphate (MOPM-Fe^3+^) membranes (Fig. S9). The successful fabrication of COF and MOPM-Fe^3+^ membranes at water–water interfaces demonstrated that the solubility of monomer in aqueous phase was the primary factor to evaluate the application university of aqueous two-phase interfacial assembly method. The aqueous solubility of monomers determined the stability of monomer solution and partition ability between aqueous two phases, which was a prerequisite for membrane fabrication. Once the selected monomers or materials could be dissolved by aqueous two phases, the structure manipulation and membrane formation could be further controlled by coordinating the diffusion–reaction rate, nucleation rate and nucleation sites, demonstrating that fabricating membranes through aqueous two-phase interfacial assembly are a kind of generic method.

### Investigation of Membrane Formation Mechanism

**Fig. 2:**
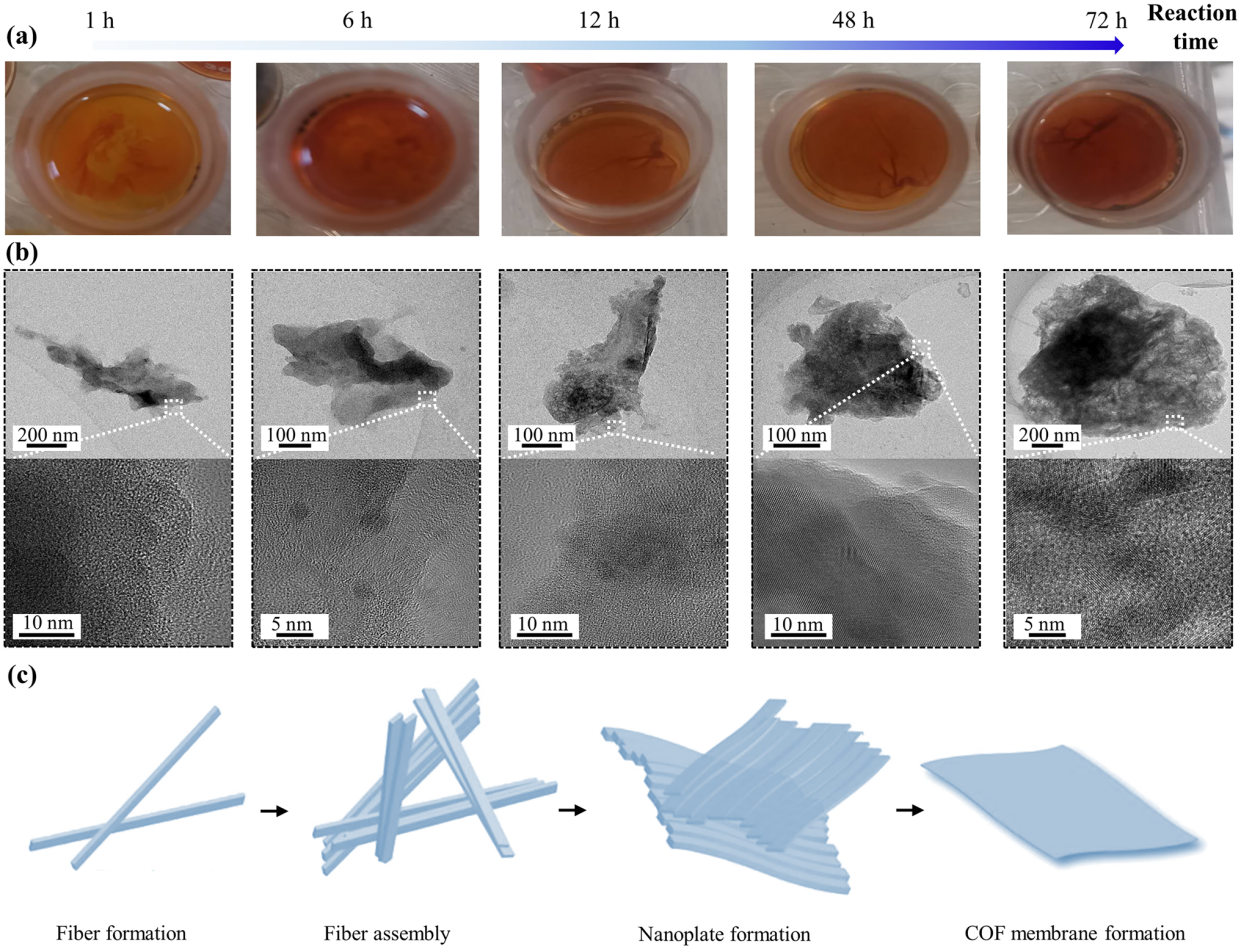
Proposed mechanism for COF membrane formation (COF-DhTG_Cl_). **a** Digital images, **b** TEM images and **c** scheme illustration of materials obtained at different stages (fiber formation, assembled fibers, and nanoplate formation) of membrane formation

To illustrate aqueous two-phase interfacial assembly, COF-DhTG_Cl_ was used as the example to investigate the structure evolution of membranes at different stage (Fig. 2). It can be found that only a few of species with fiber configuration generated after 1 h of reaction time. As the reaction time progressed to 6 h, the fiber continued to grow and assemble, forming amorphous lamellar structures with a size around 0.3 × 0.4 μm^2^. With the reaction time reaching 12 h, continuous membranes were obtained at water–water interface. At high magnification, the nanoplates did not show obvious growth, manifesting that the nanoplate had an assembly process during 6 to 12 h. Besides, at higher magnitude, the nanoplate only exhibited amorphous structures. With further increase in the reaction time to 48 and 72 h, the membranes and the corresponding nanoplates at water–water interface maintained a size around 0.4 × 0.4 μm^2^. At the same time, it can be seen that the microstructure of COF-DhTG_Cl_ membranes had large-scale staggered lattice stripes. The large-scale staggered lattice stripes agreed well with the result of GIWAXS spectrum, showing the disordered assembly of COF units. Owing to the semi-confined interface zone (tens of micrometer) [28], the formed COF units tended to grow randomly to render nondirectional channels inside membranes. Moreover, the appearance of lattice stripes also indicated that the overall crystallinity of the membrane structure has been significantly improved after a long reaction time.

**Fig. 3:**
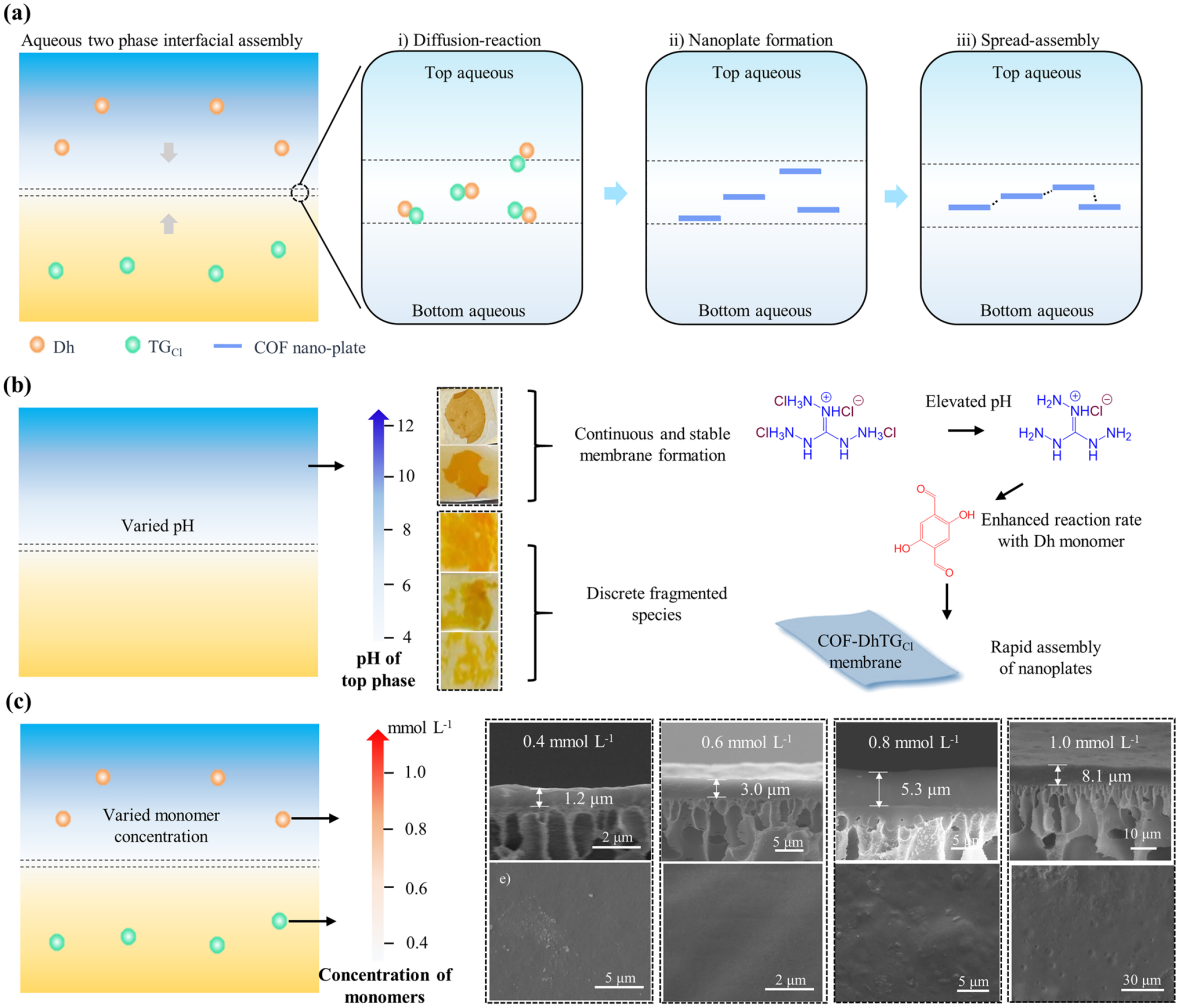
Structural evolution of COF-DhTG_Cl_ membranes. **a** Scheme of interfacial assembly mechanism. **b** Membrane formation behavior under different pH of top phase. **c** Membrane morphologies under different monomer concentration

For interfacial assembly (or interfacial polymerization) of crystalline polymer membranes, structural evolution usually undergone the following steps (Fig. 3a): (i) Diffusion–reaction. The monomers dissolved in two phases diffused oppositely and met at interface zone to proceed the reversible reaction process. (ii) Nanoplate formation. The monomers reacted to form crystalline nanoplates. The formation rates and quality of crystalline nanoplates were synergistically controlled by kinetic process (determined by the partition of monomers toward the reverse phase) and thermodynamic process (defined by bonding type between two monomers). (iii) Spread-assembly. The formed nanoplates were anchored by interface zone, spread and assembled within interface zone. Particularly, for interfacial assembly at ATPS, it was worthy to note the polymers (PEG or Dex) partitioned between aqueous two phases had non-negligible effects on membrane formation. During nanoplate formation, the interactions between monomers and polymers (PEG or Dex) influenced the diffusion and contact of monomers, which limited the diffusion process and promoted the equilibrium of diffusion–reaction process, thus conferring mild nanoplate formation environments. During spread-assembly, the polymers (PEG or Dex) restricted the efficient contact among the formed COF units due to the steric hindrance. Therefore, the polymer may remain in the bulk of COF membranes and need adequate washing to remove any residual. In this work, to demonstrate the membrane formation process, we investigated the effect of pH and monomer concentration on COF-DhTG_Cl_ membranes. Due to interfacial tension reflected the interactions among polymers (PEG or Dex) and the synergy effect of polymers (PEG or Dex) on structural evolution of membranes, we selected interfacial tension as the critical factor to investigate the membrane formation process. Figure 3b shows the growth of COF-DhTG_Cl_ membranes under different pH, where membrane formation was sensitive to the variation of pH in top phase. When pH was less than or equal to 8.0, there was only fragmented species at water–water interface. In contrast, when pH was 10 or 12, continuous membranes can be obtained at the interface, indicating that the reaction conditions are favorable for the formation of COF-DhTG_Cl_ membranes.

The pH leveraging phenomenon was attributed to the change of monomer (TG_Cl_) activity with different pH. At lower pH (< 10), the dissociation of guanidine salt into free amine monomer was inhibited [43]. As a result, the TG_Cl_ monomer diffused to the interface could not fully react with the aldehyde monomer, which was difficult for the formation of continuous membranes. At higher pH (˃10), the TG_Cl_ could be dissociated into free amine monomers to ensure the sufficient reaction with aldehyde monomers at the interface, thus contributing to the formation of continuous COF-DhTG_Cl_ membranes. The influence of reaction monomer concentration on COF-DhTG_Cl_ membranes was also explored, as shown in Fig. 3c and Table S2. With an increase in the monomer concentration from 0.4 to 1.0 mmol L^−1^, the membranes exhibited a gradually increasing thickness, ranging from 1.2 to 8.1 μm. The change trend of membrane thickness was similar to those obtained at oil–water interface. More importantly, owing to the semi-confined interface zone, the membrane formation had sufficient space to achieve the synergy of monomer reaction and fiber assembly. The membranes therefore exhibited micro-sized thickness and distinct flat-tight surface structures.

**Fig. 4:**
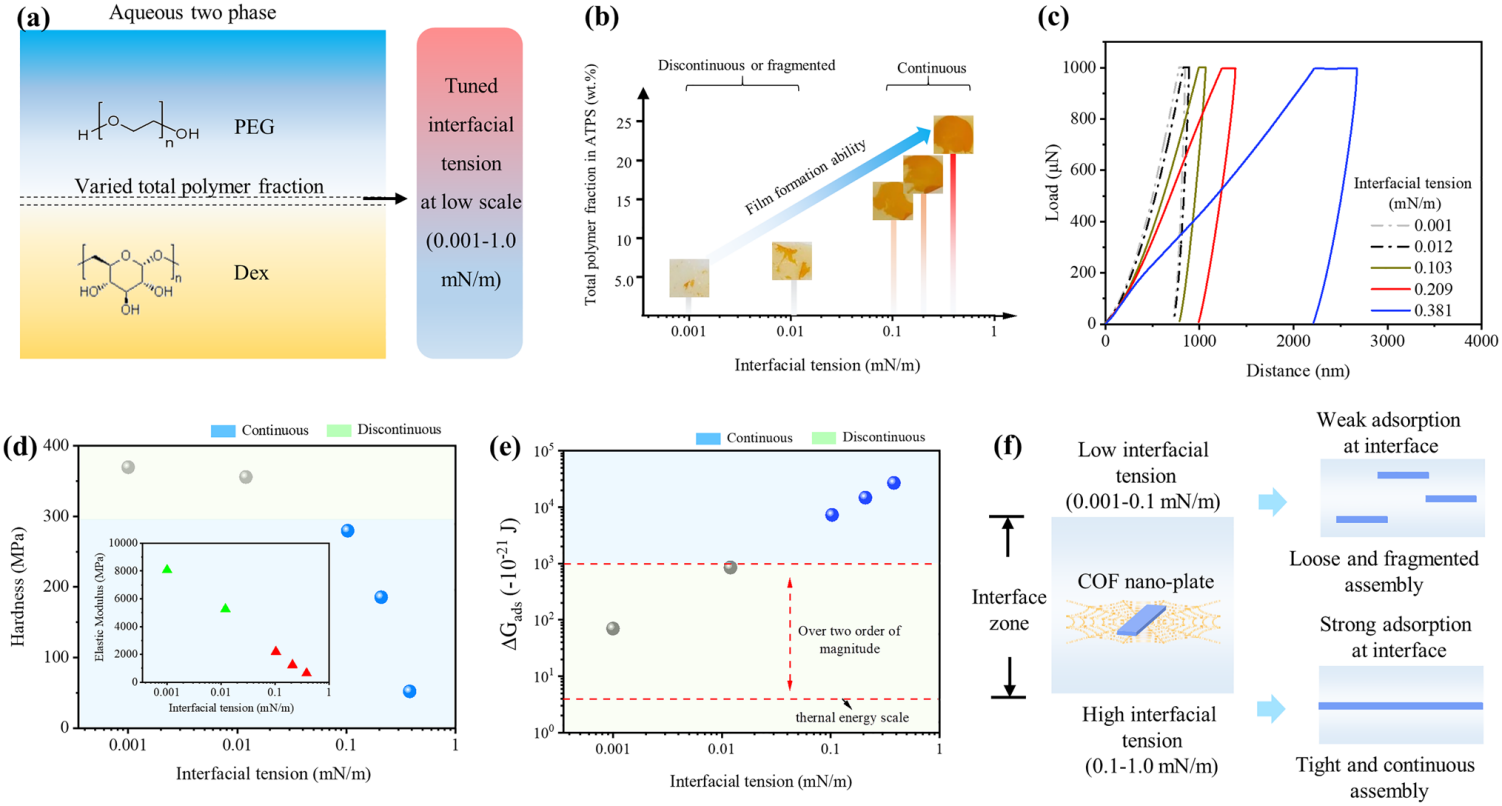
Influence of interfacial tension on formation of COF-DhTG_Cl_ membranes. **a** Scheme of tuning interfacial tension by changing total polymer fraction in aqueous two-phase system. **b** Film formation, **c** indentation depth curves, **d** hardness and elastic modulus, **e** interfacial adsorption energy and **f** spread-assembly process of COF-DhTG_Cl_ membranes under different interfacial tension

It was known that high interface tension was beneficial to the anchorage of nanoplates [44, 45], but it simultaneously led to difficult spread-assembly of nanoplates, resulting in crumple and uneven structures [32]. ATPS possessed two distinct interfacial properties, which had a low interfacial tension (0.001 mN m^−1^) that was two orders of magnitude less than that of oil–water system, and semi-confined interface zone (tens of micrometer) that was three orders of magnitude higher than that of oil–water system [28]. It was imperative to shed more light into the unique formation process of COF membranes during the aqueous two-phase interfacial assembly. Therefore, the interfacial tension was tuned by varying the relative ratio of polymer solutes in aqueous phases (Fig. 4a and Table S3) and investigated membrane structural evolution under different interfacial tension. As shown in Fig. 4b, when interfacial tension was 0.001 or 0.012 mN m^−1^, only fragmented species formed after aqueous two-phase assembly. Only a few of fragments could be obtained. In sharp contrast, when interfacial tension reaches higher value (0.103, 0.209, and 0.381 mN m ^−1^), continuous COF-DhTG_Cl_ membranes were found within the interface of ATPS, indicating interfacial tension had a significant influence on the film formation ability of COFs.

With utilizing nano-indentation analysis, the mechanical properties of COF-DhTG_Cl_ membranes (or products) were investigated under different interfacial tension. Figure 4c showed load-distance curves of membranes, where the indentation depth displayed an increasing trend with the increasing interfacial tension from 0.001 to 0.381 mN m ^−1^. Based on Oliver–Pharr method [46], the hardness and elastic modulus of membranes were calculated (Fig. 4d). Overall, both the hardness and elastic modulus of membranes decreased with the increase in interfacial tension. When interfacial tension was less than 0.1 mN m^−1^, the hardness and elastic modulus lay within 350–370 and 5,000–8,000 MPa, respectively. And discrete fragmented species formed within ATPS. When interfacial tension ranged from 0.103 to 0.381 mN m^−1^, the hardness and elastic modulus of membranes had a sharp decline, dropping from 278 and 2187 MPa at 0.103 mN m^−1^ to 50 and 636 MPa at 0.381 mN m^−1^, respectively. Noting that continuous membranes were successfully formed at higher interfacial tension (0.103–0.381 mN m^−1^), it could be speculated that higher interfacial tension contributed to the assembly among COF nanoplates and fortified the linking strength inside COF membranes. We further studied the influence of interfacial adsorption energy (varied from interfacial tension) on structural evolution of membranes. The interfacial adsorption energy of ATPS toward nanoplates could be calculated from Eq. (3):

3 $$ \Delta G_{{{\text{ads}}}} \left( {{\text{nanoplate}}} \right) = - \left( {\pi /4} \right)d^{2} \gamma \left( 1 \right. - \left. {\left| {\cos \theta } \right|} \right) $$

where *d* is the average radial size of nanoplates, which was around 300 nm (Fig. 1d) and had negligible growth with increased reaction time; *γ* was the interfacial tension of ATPS; *θ* was the contact angle between nanoplates and interface. It was reported that *θ* could be regarded as 90° under static conditions [47]. Accordingly, the relationship among interfacial adsorption energy, interfacial tension and film formation ability was developed (Fig. 4e). When interfacial tension was 0.001 and 0.012 mN m^−1^, the interfacial adsorption was 7.0 × 10^–20^ and 8.5 × 10^–19^ J. At these conditions, ATPS could not provide stable adsorption-anchorage sites for assembly of nanoplates, resulting in discrete distribution of fragments inside the interfacial tension rather than continuous membranes (top-right of Fig. 4f). In contrast, when interfacial was higher than 0.1 mN m ^−1^, the interfacial adsorption energy toward nanoplates exceeded 2–4 orders of magnitude than thermal scale barrier (*k*_*B*_*T* ≈ 4.0 × 10^–21^ J), which conferred favorable microenvironments for the adsorption-anchorage of nanoplates in interface zone and generated continuous and tight COF membranes (bottom-right of Fig. 4f).

### Desalination Performances of Membranes

**Fig. 5:**
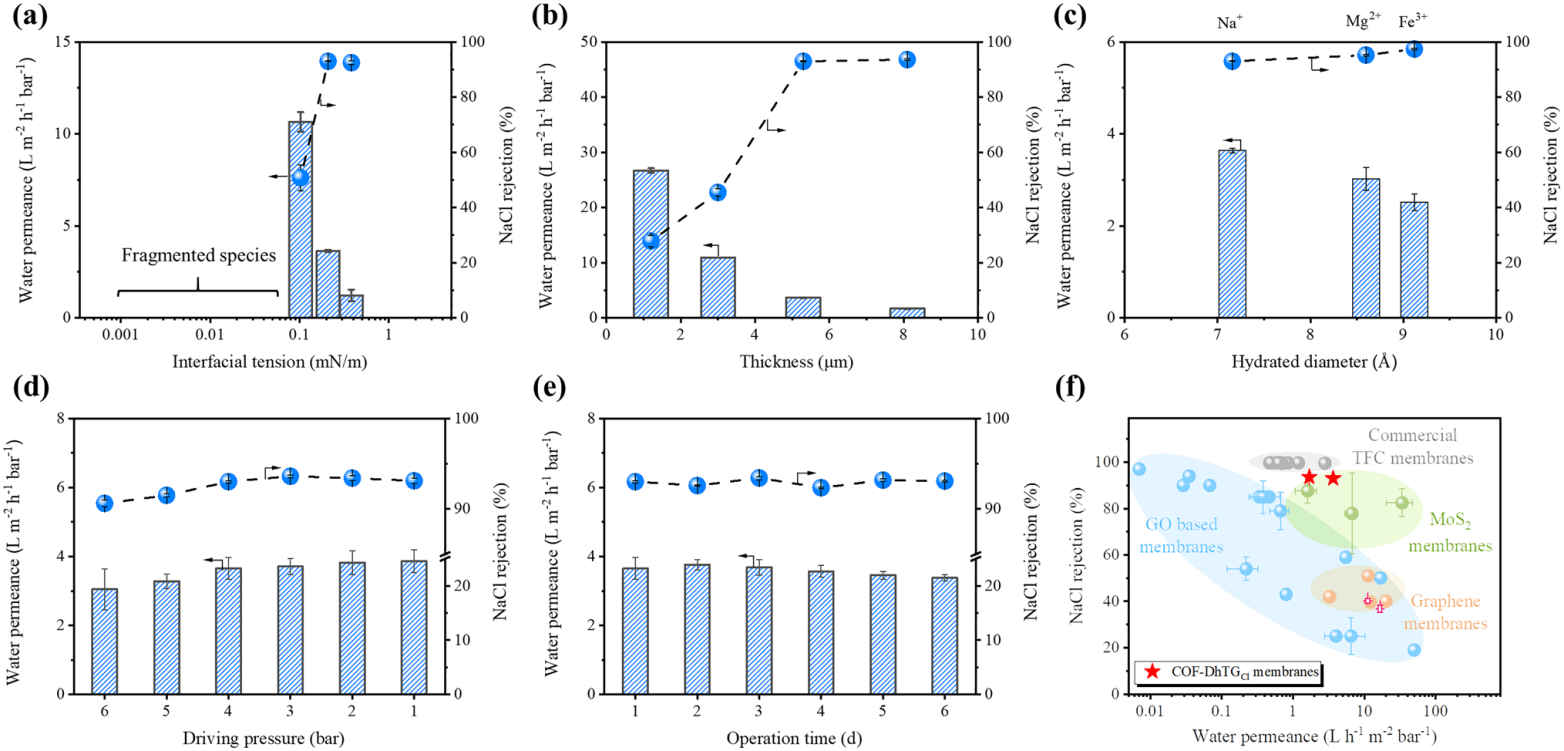
Desalination performances of COF-DhTG_Cl_ membranes. **a** Membranes prepared by different interfacial tension, **b** membranes with different thickness, **c** membranes undergone different salt solution, **d** varied driving pressure test, **e** long-term operation test and **f** comparison of desalination performances with those reported in the literatures

The desalination performances of membranes were evaluated using a cross-flow configuration (Fig. S10). The feed side was filled with 1,000 ppm NaCl aqueous solution, and the pressure was set as 4.0 bar during operation. Figure 5a showed the desalination performances of COF-DhTG_Cl_ membranes prepared by different interfacial tension. Due to fragmented species formed under 0.001 and 0.012 mN m^−1^, the corresponding performances could not be detected. When interfacial tension increased from 0.103 to 0.209mN m^−1^, the water permeance of membranes decreased from 10.7 ± 0.5 to 3.6 ± 0.5 L m^−2^ h^−1^ bar^−1^, and the corresponding NaCl rejection varied from 50.8 ± 4.7 to 93.0 ± 0.2. Moreover, with further increase in interfacial tension to 0.381 mN m^−1^, the water permeance declined to 1.2 ± 0.5 L m^−2^ h^−1^ bar^−1^, and the NaCl rejection maintained at 92.5 ± 0.8. The desalination performances were affected by continuity (or density), channel size and charge property of COF-DhTG_Cl_ membranes. As mentioned above, lower interfacial tension led to the loose or fragmented membranes (Fig. 4), which was favorable for the transport of target species unfavorable for the rejection performances of membranes. As a result, COF-DhTG_Cl_ membranes prepared by 0.103 mN m ^−1^ of interfacial tension had higher water permeance and lower NaCl rejection. In contrast, membranes with tighter structures (prepared by 0.209 and 0.381 mN m^−1^) efficiently utilized confined channel (0.56 nm) and positive charge, showing superior NaCl rejection (~ 93%). It was noting that COF-DhTG_Cl_ membranes had a relative low water permeance compared with anionic COF membranes, which was mainly due to that the poor hydrophilicity of COF-DhTG_Cl_ membranes (Fig. S7) increased transport resistance of water molecules [48].

The influence of membrane thickness on desalination performances was investigated (Fig. 5b). The increased membrane thickness (from 1.2 to 8.1 μm) lowered the water permeance gradually from 26.7 ± 0.4 to 1.7 ± 0.1 L m^−2^ h^−1^ bar^−1^, along with the increased NaCl rejection ranging from 27.9 ± 2.1 to 93.6 ± 0.3. This result confirmed the importance of continuity (or density) on membrane performances. The increased membrane thickness could improve the confined function of membrane channel, thus conferring membranes with high NaCl rejection. Furthermore, the types of salt (single NaCl, MgCl_2_ and FeCl_3_) in feed side was varied to evaluate the desalination performances of COF-DhTG_Cl_ membranes (Fig. 5c). With tuning the feed side from NaCl to MgCl_2_, and reaching FeCl_3_, the water permeance decreased from 3.64 to 2.51 L m^−2^ h^−1^ bar^−1^, along with salt rejection increased from 93.0 to 97.4%. The considerable salt rejection performances of COF-DhTG_Cl_ membranes were due to the synergy of electrostatic repulsion and size sieving. On the one hand, the increased charge of cations could fortify electrostatic repulsion between cations and membranes, conferring membranes with high salt rejection. On the other hand, the hydrated diameter of Na^+^, Mg^2+^ and Fe^3+^ was 7.2, 8.6 and 9.1 Å, respectively [49]. The increased hydrated diameter considerably enhanced the sieving function of membranes, leading to higher salt rejection and lower water permeance.

Desalination performances under different driving pressure was also evaluated. As shown in Fig. 5d, with the driving pressure decreased from 6.0 to 1.0 bar, both the water permeance and NaCl rejection of COF-DhTG_Cl_ membranes showed slight change, which were 3.1–3.9 L m^−2^ h^−1^ bar^−1^ and 91–93%, respectively. The steady desalination performances under varied driving pressure were attributed to the robust frameworks, which enabled membranes with strong resistance toward adverse structural evolution. Similar results were observed in long-term operation test (Fig. 5e), where COF-DhTG_Cl_ membranes exhibited steady water permeance (keeping at 3.3–3.7 L m^−2^ h^−1^ bar^−1^) and NaCl rejection (maintaining at 92–93%). The superior performances under different driving pressure and operation time ensured COF-DhTG_Cl_ membranes alternative candidates for potential diverse applications. The desalination performances (NaCl rejection) of COF-DhTG_Cl_ membranes were used to compare with current reverse osmosis/nanofiltration/forward osmosis membranes (Fig. 5f and Table S4). It can be seen that the performances of COF-DhTG_Cl_ membranes lay in the up-right side. The 93%–93.6% NaCl rejection of COF-DhTG_Cl_ membranes exceeded those of graphene oxide (GO), MoS_2_ and graphene membranes, and close to those (~ 99%) of commercial thin film composite (TFC) membranes. Meanwhile, COF-DhTG_Cl_ membranes exhibited 1.7–3.7 L m^−2^ h^−1^ bar^−1^ of water permeance, which was about 2–4 times of commercial TFC membranes, demonstrating the superior desalination performances of COF-DhTG_Cl_ membranes.

## Conclusions

In summary, we developed an aqueous two-phase interfacial assembly method to fabricate COF membranes, establishing a solvent-free platform for fabricating advanced membranes. In particular, interfacial tension was found to exert pronounced effect on structural evolution of COF membranes. The moderate interfacial tension (0.1–1.0 mN m^−1^) led to the tight and stable assembly of COF membranes. The resultant COF-DhTG_Cl_ membranes exhibited NaCl rejection of 93.0%–93.6% and water permeance of 1.7–3.7 Lm^−2^ h^−1^ bar^−1^. Our work is the pioneering attempt of fabricating membranes at water–water interface, which may represent a technological breakthrough in green membrane manufacturing method.

## Supplementary Information

Below is the link to the electronic supplementary material.

Supplementary file1 (PDF 970 KB)
