# Supplementary material for: Aqueous Two-Phase Interfacial Assembly of COF Membranes for Water Desalination
Source: Nanomicro Lett. 2022 Nov 9;14:216. doi: 10.1007/s40820-022-00968-5 (PMC9646690; doi:10.1007/s40820-022-00968-5)
Supplement: Supplementary file 1 — Supplementary file1 (PDF 970 KB) [file 40820_2022_968_MOESM1_ESM.pdf]

Supporting Information for

Aqueous Two-Phase Interfacial Assembly of COF Membranes for Water Desalination

Hongjian Wang[1, 2, ‡], Jiashuai Zhao[1, 2, ‡], Yang Li[3], Yu Cao[1, 2], Ziting Zhu[1, 2], Meidi Wang[1, 2], Runnan Zhang[1, 2, 4], Fusheng Pan[1, 2, 4, *] and Zhongyi Jiang[1, 2, 4, *]

[1]Key Laboratory for Green Chemical Technology of Ministry of Education, School of Chemical Engineering and Technology, Tianjin University, Tianjin 300072, P. R. China

[2]Haihe Laboratory of Sustainable Chemical Transformations, Tianjin 300192 , P. R. China

[3]Department of Chemistry, Tianjin Key Laboratory of Molecular Optoelectronic Science, Tianjin University, Tianjin 300072, P. R. China

[4]Joint School of National University of Singapore and Tianjin University, International Campus of Tianjin University Binhai New City, Fuzhou 350207, P. R. China

[‡]Hongjian Wang and Jiashuai Zhao contributed equally to this work.

*Corresponding authors. E-mail: zhyjiang@tju.edu.cn (Zhongyi Jiang), fspan@tju.edu.cn (Fusheng Pan)

Supplementary Figures and Tables

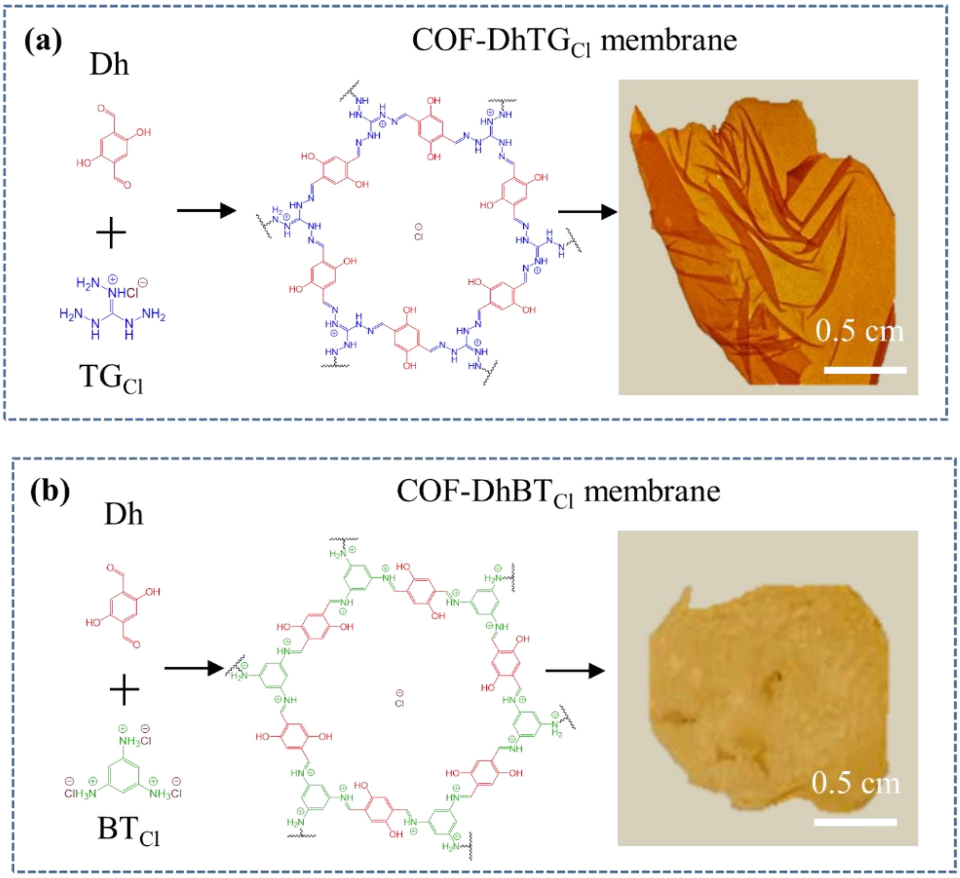

Fig. S1 Schematic illustration and digital photos of **a** COF-DhTG$_{Cl}$ and **b** COF-DhBT$_{Cl}$ membranes

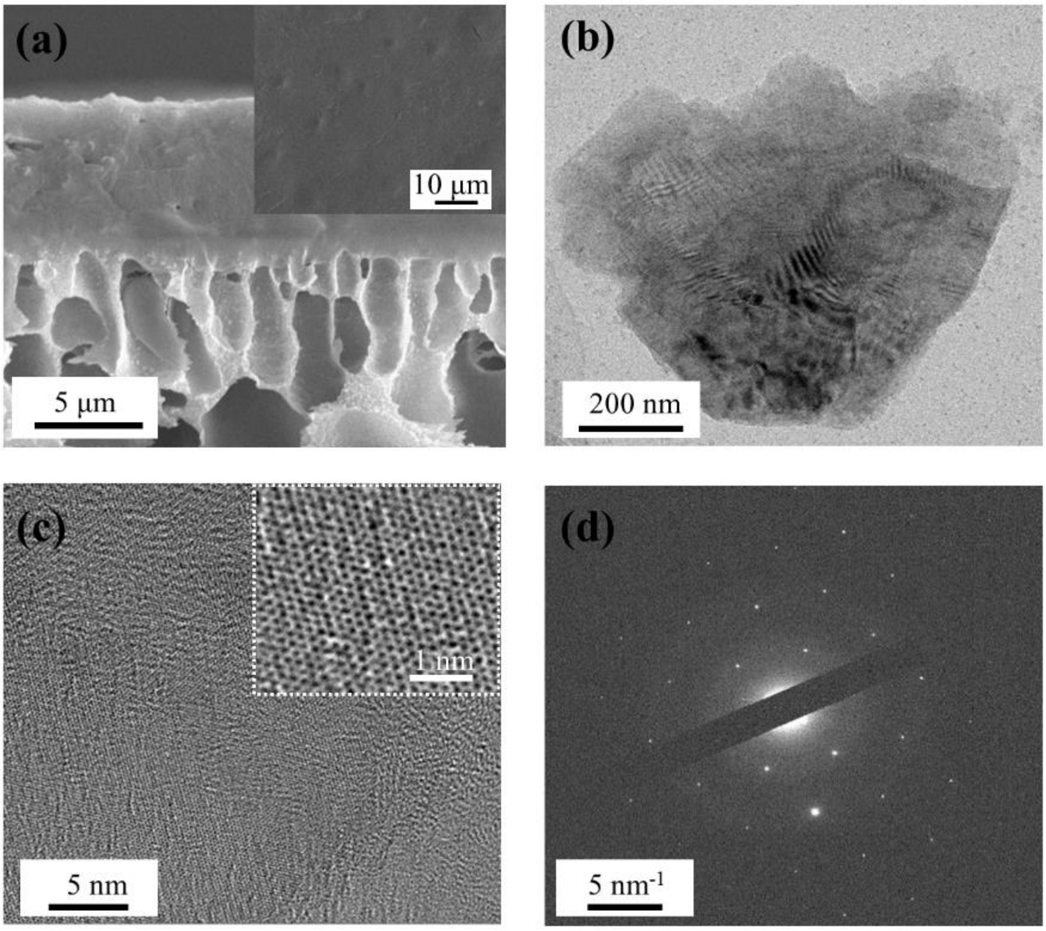

Fig. S2 a SEM, **b-c** TEM and **d** SAED measurements on COF-DhBT$_{Cl}$ membrane

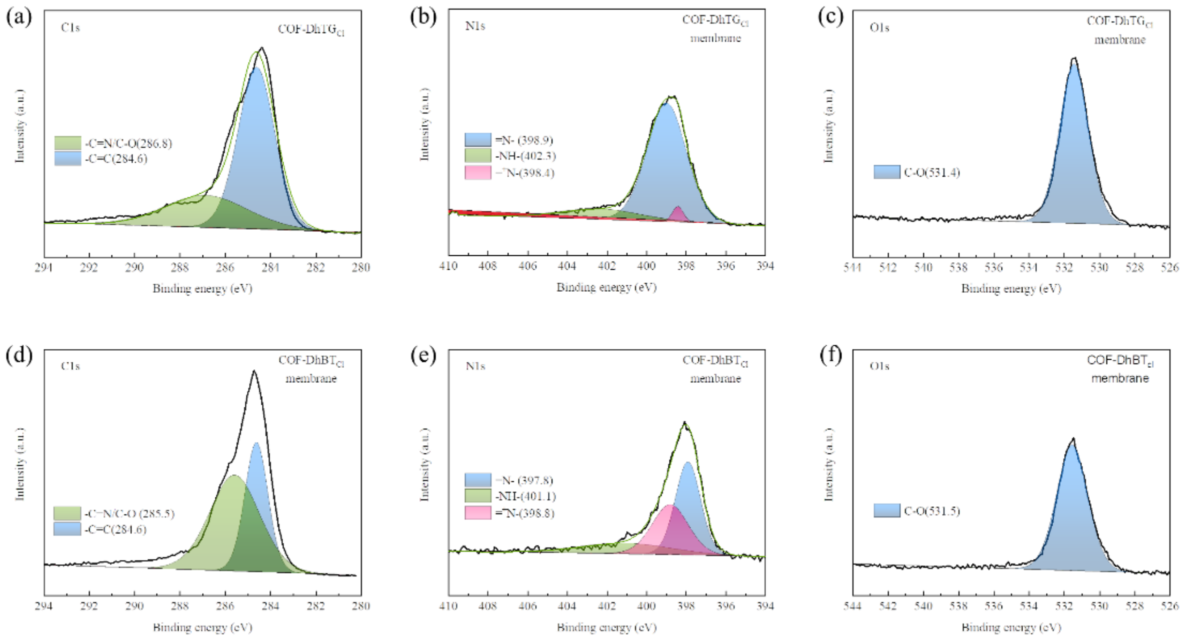

Fig. S3 XPS spectra of COF membranes. **a** C 1s, **b** N 1s and **c** O 1s spectra of COF-DhTG$_{Cl}$ membrane. **d** C 1s, **e** N 1s and **f** O 1s spectra of COF-DhBT$_{Cl}$ membrane

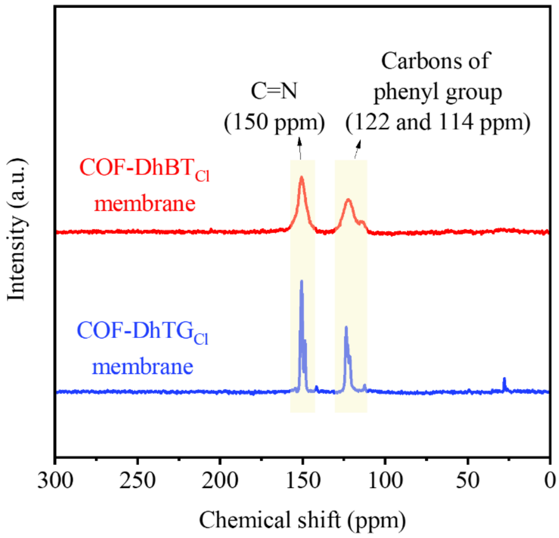

Fig. S4 ssNMR spectra of COF-DhTG$_{Cl}$ and COF-DhBT$_{Cl}$ membranes

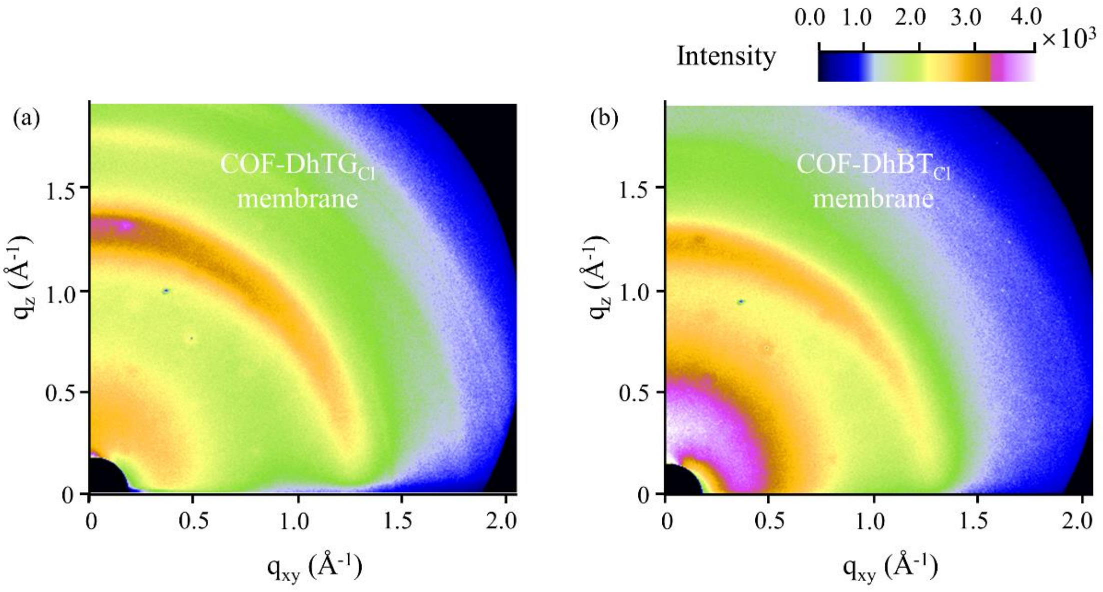

Fig. S5 2D-GIWAXS spectra of **a** COF-DhTG$_{Cl}$ and **b** COF-DhBT$_{Cl}$ membranes

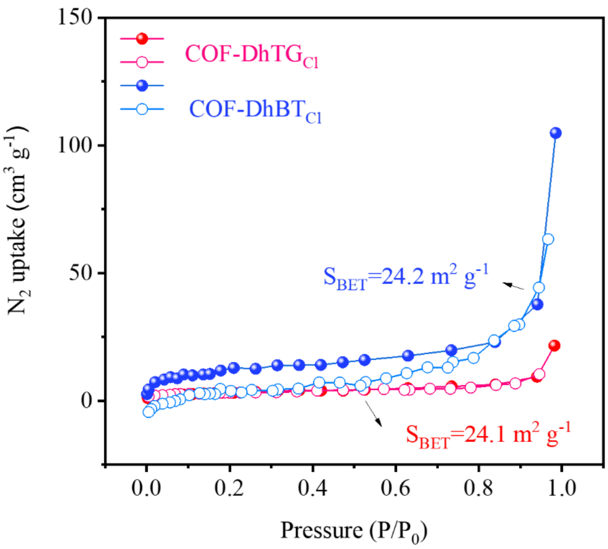

Fig. S6 N_2 adsorption measurement of COF membranes

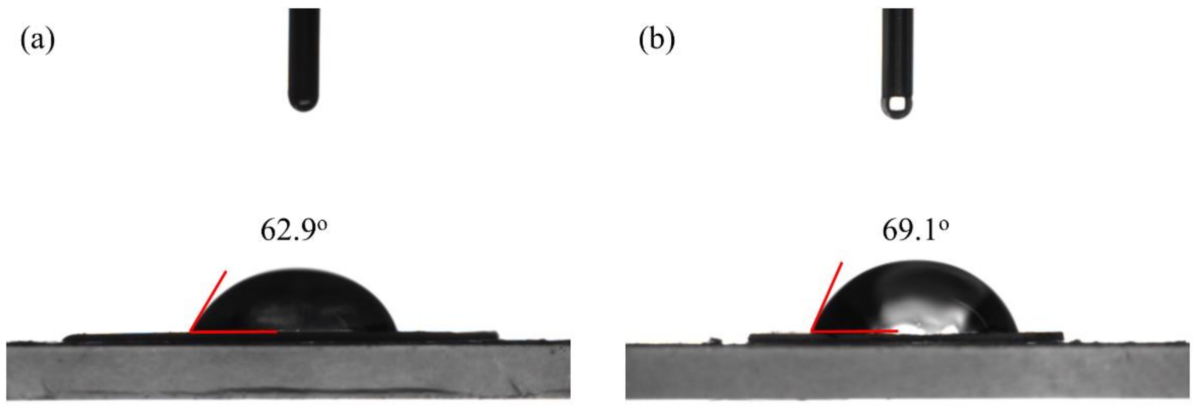

Fig. S7 WCA measurement of **a** COF-DhTG$_{Cl}$ and **b** COF-DhBT$_{Cl}$ membranes, respectively

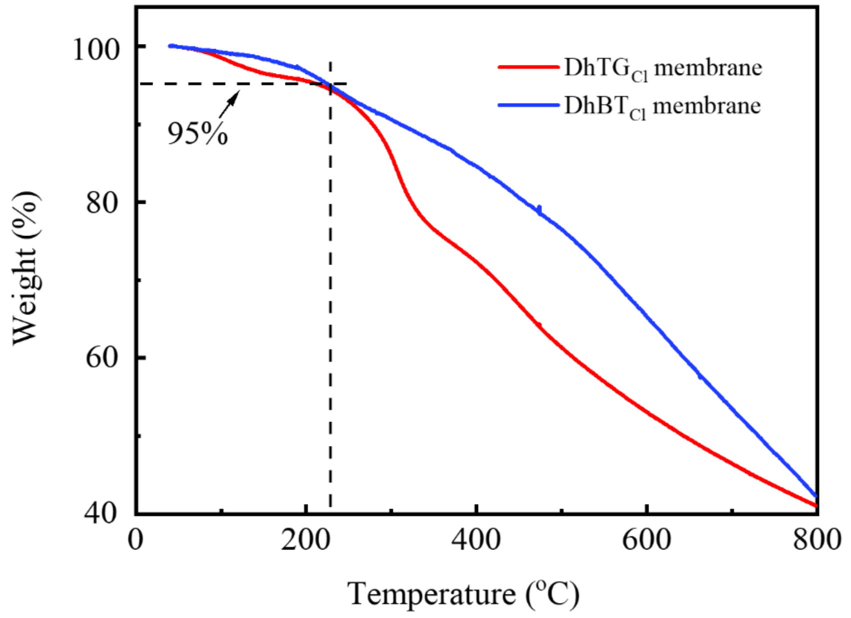

Fig. S8 TGA measurement of COF membranes

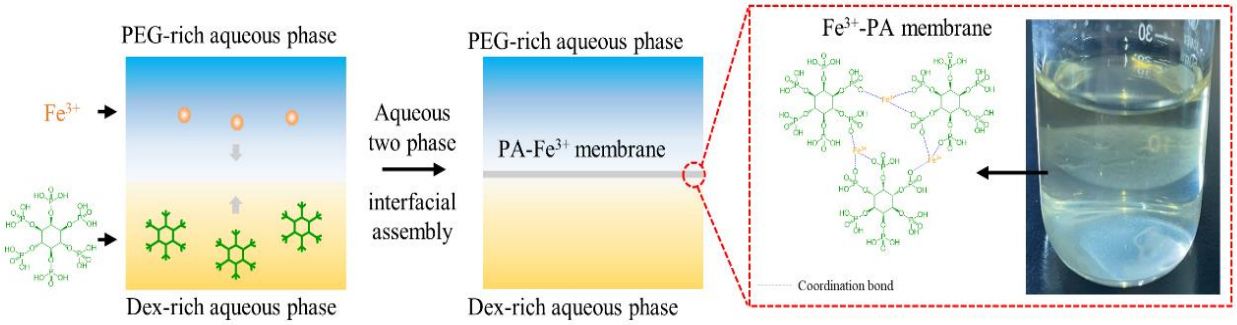

Fig. S9 Scheme illustration of fabricating metal organic polymer (metal-organophosphate) membranes using aqueous two-phase interfacial assembly

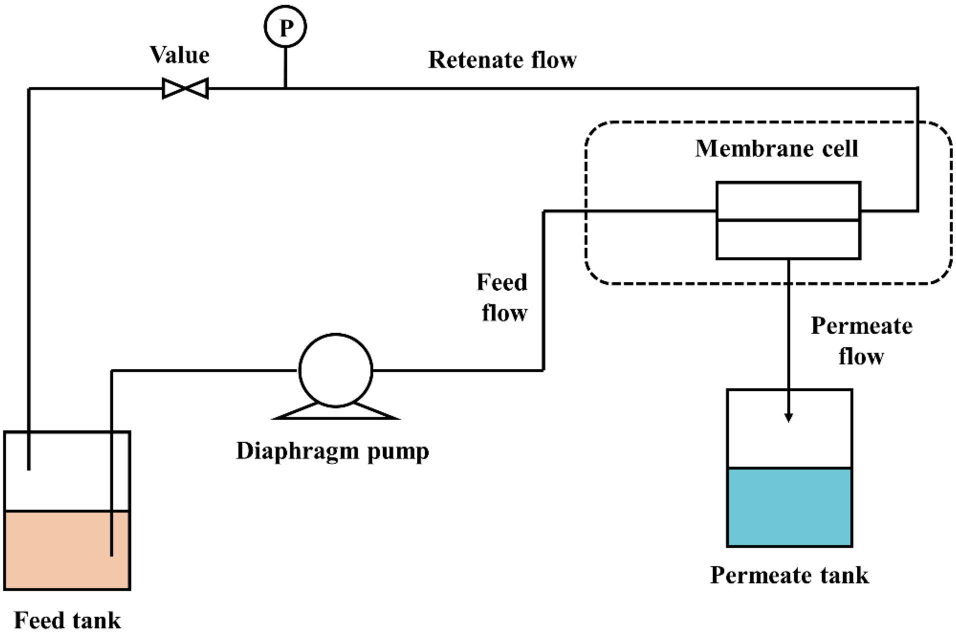

Fig. S10 Apparatus of nanofiltration desalination with cross-flow configuration

Table S1 Zeta potential of COF membranes

Membrane	Zeta potential (ξ, mV)
COF-DhTG$_{Cl}$	20.1 ± 1.2
COF-DhBT$_{Cl}$	9.8 ± 1.8

Table S2 Monomer concentration in the recipe for COF-DhTG$_{Cl}$ membrane fabrication

Name	Dh concentration (μmol mL^{-1})	TG$_{Cl}$ concentration (μmol mL^{-1})
M1	0.4	0.25
M2	0.6	0.4
M3	0.8	0.5
M4	1.0	0.7

Table S3 Phase composition and interfacial properties [S1, S2] of ATPS in this study

Name	PEG fraction in solution A (wt%)	DEx fraction insolution B (wt%)	Total polymer fraction (wt%)	Interfacial tension (mN m^{-1})
1	2.1	2.6	2.30	0.001
2	5.0	6.4	5.62	0.012
3	10	16	12.6	0.103
4	15	19.2	16.3	0.209
5	20	25.6	21.4	0.381

Table S4 Nanofiltration/reverse osmosis/forward osmosis desalination (NaCl rejection) performances of membranes in literatures

Membrane	Filtration method	Pressure (bar)	Water permeation (L m^2 h^{-1} bar^{-1})	NaCl rejection (%)	Refs.
Graphene/GO	Cross flow	50	0.34 (± 0.1)	85 (± 2)	[S3]
Graphene/GO	Cross flow	50	0.22 (± 0.1)	54 (± 5)	[S3]
Graphene/GO	Cross flow	50	0.46 (± 0.2)	85 (± 1)	[S3]
Graphene/GO	Cross flow	50	0.38 (± 0.1)	85 (± 7)	[S3]
Graphene/GO	Cross flow	50	0.44 (± 0.02)	83 (± 5)	[S3]
Graphene/GO	Cross flow	50	0.67 (± 0.02)	79 (± 8)	[S3]
Laminated GO	Dead end	2	4	25	[S4]
GO/TMC	Dead end	3.4	~50	19	[S5]
GO/CNT	Dead end	5	5.5	59	[S6]
GO/PECs	Cross flow	5	0.80	43	[S7]
GO	Dead end	1.5	16.9	50.1	[S8]
GO	Forward osmosis	0.28	0.068 (± 0.007)	90	[S9]
GO	Forward osmosis	0.28	0.029 (± 0.005)	90	[S9]
GO/graphene	Forward osmosis	1	0.035	~94	[S10]
GO/graphene	Forward osmosis	5	0.007	97	[S10]
GO	Forward osmosis	5	0.0084	60	[S10]
Graphene	Dead end	1	20	40	[S11]
Graphene/CNT	Dead end	5	11.3	51	[S6]
Graphene/CNT	Cross flow	5	12.1	39.6	[S6]
Graphene	Dead end	5	3.26	42	[S11]
MoS_2	Dead end	9	33.7 (± 13.5)	82.5 (± 6)	[S12]
MoS_2	Dead end	9	1.6 (± 0.53)	87.6 (± 5.2)	[S12]
MoS_2	Dead end	9	6.7 (± 1.35)	77.9 (± 17.4)	[S12]

Supplementary References

[S1] E. Atefi, J.A. Mann, H. Tavana, Ultralow interfacial tensions of aqueous two-phase systems measured using drop shape. Langmuir **30**(32), 9691-9699 (2014). https://doi.org/10.1021/la500930x

[S2] S.D. Hann, K.J. Stebe, D. Lee, All-aqueous assemblies via interfacial complexation: toward artificial cell and microniche development. Langmuir **33**(39), 10107-10117 (2017). https://doi.org/10.1021/acs.langmuir.7b02237

[S3] A. Morelos-Gomez, R. Cruz-Silva, H. Muramatsu, J. Ortiz-Medina, T. Araki et al., Effective NaCl and dye rejection of hybrid graphene oxide/graphene layered membranes. Nat. Nanotechnol. **12**(11), 1083-1088 (2017). https://doi.org/10.1038/nnano.2017.160

[S4] Q. Yang, Y. Su, C. Chi, C.T. Cherian, K. Huang et al., Ultrathin graphene-based membrane with precise molecular sieving and ultrafast solvent permeation. Nat. Mater. **16**(12), 1198-1202 (2017). https://doi.org/10.1038/nmat5025

[S5] M. Hu, B. Mi, Enabling graphene oxide nanosheets as water separation membranes. Environ. Sci. Technol. **47**(8), 3715-3723 (2013). https://doi.org/10.1021/es400571g

[S6] Y. Han, Y. Jiang, C. Gao, High-flux graphene oxide nanofiltration membrane intercalated by carbon nanotubes. ACS Appl. Mater. Interfaces **7**(15), 8147-8155 (2015). https://doi.org/10.1021/acsami.5b00986

[S7] N. Wang, S. Ji, G. Zhang, J. Li, L. Wang, Self-assembly of graphene oxide and polyelectrolyte complex nanohybrid membranes for nanofiltration and pervaporation. Chem. Eng. J. **213**, 318-329 (2012). https://doi.org/10.1016/j.cej.2012.09.080

[S8] G. Zhao, R. Hu, X. Zhao, Y. He, H. Zhu, High flux nanofiltration membranes prepared with a graphene oxide homo-structure. J. Membr. Sci. **585**, 29-37 (2019). https://doi.org/10.1016/j.memsci.2019.05.028

[S9] L. Chen, G. Shi, J. Shen, B. Peng, B. Zhang et al., Ion sieving in graphene oxide membranes via cationic control of interlayer spacing. Nature **550**(7676), 380-383 (2017). https://doi.org/10.1038/nature24044

[S10] J. Abraham, K.S. Vasu, C.D. Williams, K. Gopinadhan, Y. Su et al., Tunable sieving of ions using graphene oxide membranes. Nat. Nanotechnol. **12**(6), 546-550 (2017). https://doi.org/10.1038/nnano.2017.21

[S11] Y. Han, Z. Xu, C. Gao, Ultrathin graphene nanofiltration membrane for water purification. Adv. Funct. Mater. **23**(29), 3693-3700 (2013). https://doi.org/10.1002/adfm.201202601

[S12] L. Ries, E. Petit, T. Michel, C.C. Diogo, C. Gervais et al., Enhanced sieving from exfoliated MoS_2 membranes via covalent functionalization. Nat. Mater. **18**(10), 1112-1117 (2019). https://doi.org/10.1038/s41563-019-0464-7
